# Supplementary material for: Agonist immunotherapy restores T cell function following MEK inhibition improving efficacy in breast cancer
Source: Nat Commun. 2017 Sep 19;8:606. doi: 10.1038/s41467-017-00728-9 (PMC5605577; doi:10.1038/s41467-017-00728-9)
Supplement: Supplementary file 1 — Supplementary Information [file 41467_2017_728_MOESM1_ESM.pdf]

Description of Supplementary Files

File Name: Supplementary Information
Description: Supplementary Figures

File Name: Supplementary Data 1
Description: Differential gene expression analysis following MEKi. Differentially expressed genes in 4T1Ch9 tumors following 7 days of vehicle versus MEKi treatment in vivo. False discovery rate was set at <10%. Top genes were selected based on a P value of <0.05 and a fold change of >1.5.

File Name: Supplementary Data 2
Description: GeneGo pathway analysis of differentially expressed genes of 4T1Ch9 MEKi treated tumors. MEKi treatment was administered for 7 days in the 4T1Ch9 tumors in vivo and differential expression was undertaken on vehicle versus MEKi treated tumor RNA ex vivo. False discovery rate was set at <10%. Top genes were selected based on a P value of <0.05 and a fold change of >1.5.

File Name: Supplementary Data 3
Description: Gene Set Enrichment Analysis following MEKi - Immune Response. Analysis of differentially expressed genes of 4T1Ch9 MEKi treated tumors . MEKi treatment was administered for 7 days in the 4T1Ch9 tumors in vivo and differential expression was undertaken on vehicle versus MEKi treated tumor RNA ex vivo. False discovery rate was set at <10%. Top genes were selected based on a P value of <0.05 and a fold change of >1.5.

File Name: Supplementary Data 4
Description: Gene Set Enrichment Analysis following MEKi - T cell activation.Analysis of differentially expressed genes of 4T1Ch9 MEKi treated tumors . MEKi treatment was administered for 7 days in the 4T1Ch9 tumors in vivo and differential expression was undertaken on vehicle versus MEKi treated tumor RNA ex vivo. False discovery rate was set at <10%. Top genes were selected based on a P value of <0.05 and a fold change of >1.5.

File Name: Supplementary Data 5
Description: Gene Set Enrichment Analysis following MEKi - JNK pathway upregulation. Analysis of differentially expressed genes of 4T1Ch9 MEKi treated tumors . MEKi treatment was administered for 7 days in the 4T1Ch9 tumors in vivo and differential expression was undertaken on vehicle versus MEKi treated tumor RNA ex vivo. False discovery rate was set at <10%. Top genes were selected based on a P value of <0.05 and a fold change of >1.5.

File Name: Supplementary Data 6
Description: Gene Set Enrichment Analysis following MEKi - NFkB pathway activation. Analysis of differentially expressed genes of 4T1Ch9 MEKi treated tumors . MEKi treatment was administered for 7 days in the 4T1Ch9 tumors in vivo and differential expression was undertaken on vehicle versus MEKi treated tumor RNA ex vivo. False discovery rate was set at <10%. Top genes were selected based on a P value of <0.05 and a fold change of >1.5.

File Name: Peer Review File

AT3ova

A

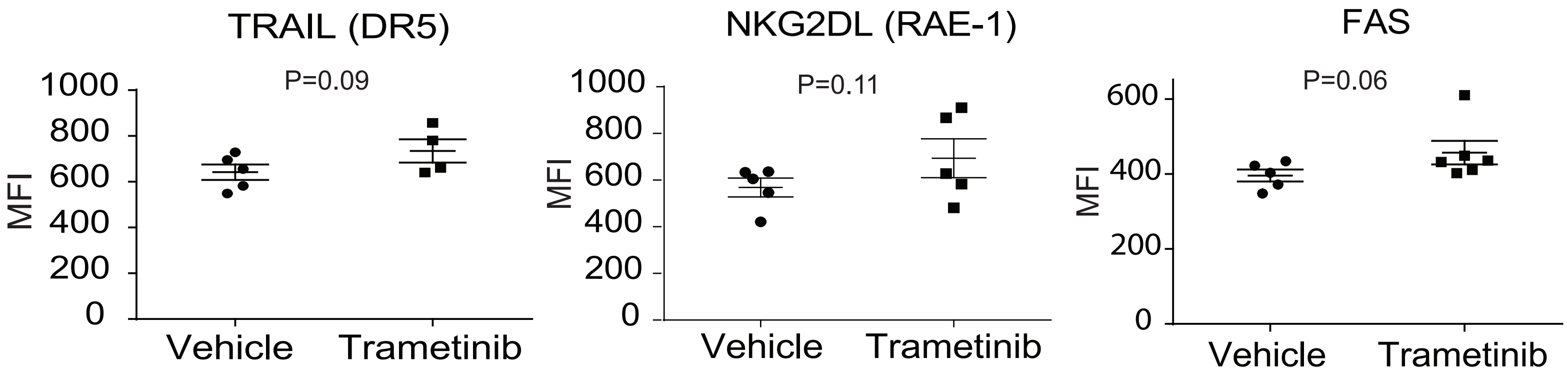

TRAIL (DR5)
P=0.09
1000
800
600
400
200
0
MFI
Vehicle
Trametinib
NKG2DL (RAE-1)
P=0.11
1000
800
600
400
200
0
MFI
Vehicle
Trametinib
FAS
P=0.06
600
400
200
0
MFI
Vehicle
Trametinib

Supplementary Figure 1. MEK inhibition increases tumor immunogenicity markers on AT3ova and 4T1Ch9 TNBC tumors in vivo. Mice (n=5 per group) bearing established AT3ova tumors were treated with vehicle (PEG 400/solutol) or trametinib (1mg/kg/daily) for 4 days. FACS analysis of TRAIL(DR5), NKG2DL(RAE-1) and Fas expression (mean fluorescence index; MFI) on the AT3ova tumors was undertaken. Data is presented as mean ± SEM. P values represent unpaired t-tests and post-hoc Fishers' LSD tests. *P<0.05, **P<0.01, ***P<0.001, ****P<0.0001.

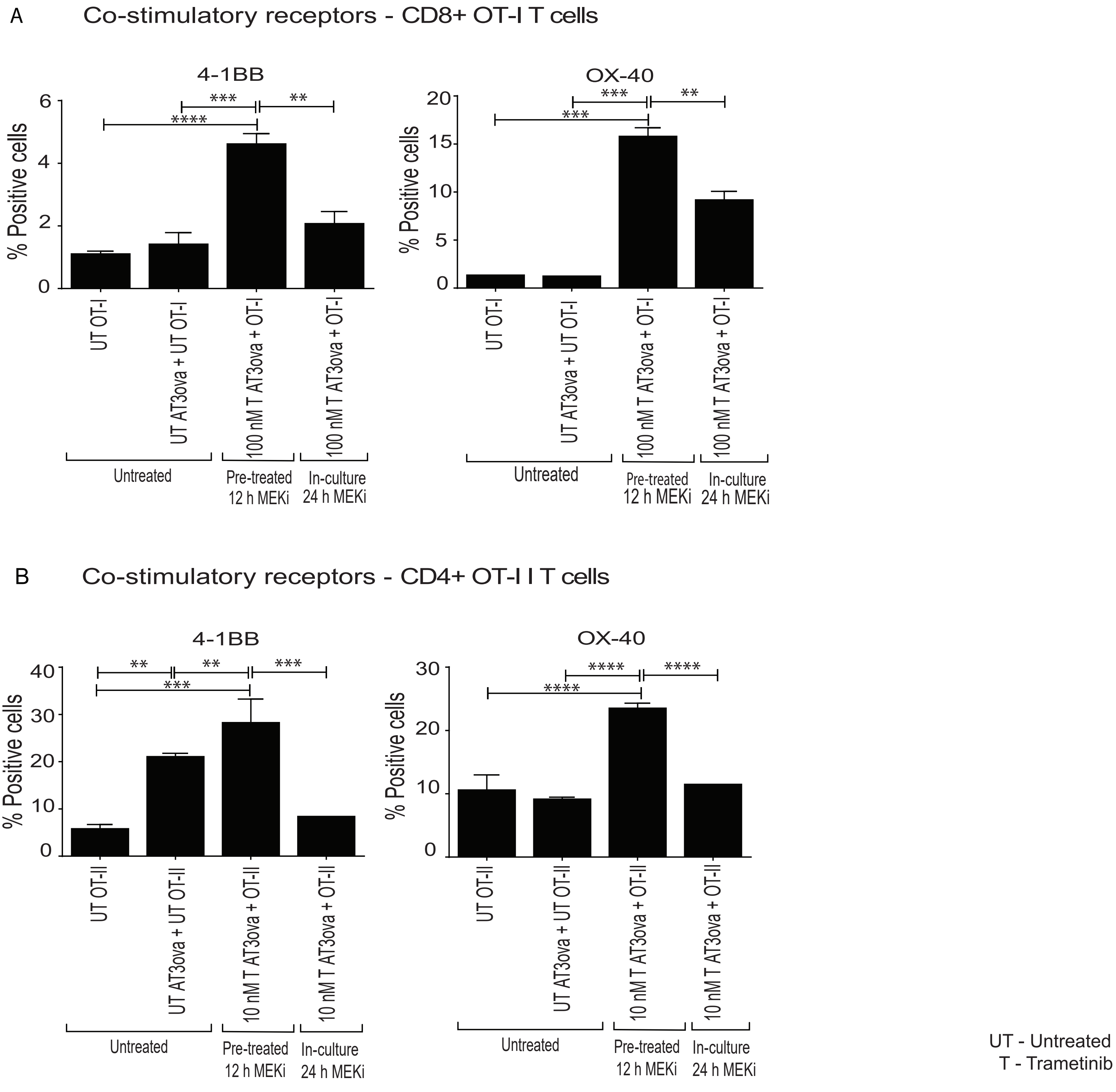
A
Co-stimulatory receptors - CD8+ OT-I T cells
4-1BB
OX-40
% Positive cells
UT OT-I
UT AT3ova + UT OT-I
100 nM T AT3ova + OT-I
Untreated
Pre-treated 12 h MEKi
In-culture 24 h MEKi
B
Co-stimulatory receptors - CD4+ OT-I I T cells
4-1BB
OX-40
% Positive cells
UT OT-II
UT AT3ova + UT OT-II
10 nM T AT3ova + OT-II
Untreated
Pre-treated 12 h MEKi
In-culture 24 h MEKi
UT - Untreated
T - Trametinib

Supplementary Figure 2. Increased immunogenicity of tumors following MEK inhibition results in enhanced activation of co-stimulatory receptors in OT-I CD8⁺ and OT-II CD4⁺T cells*.** OT-I or OT-II cells were co-cultured with AT3ova cells that were either pre-treated with trametinib (100nM, 10nM) for 12 hours or trametinib (100nM, 10nM) was added directly to the 24 hour co-culture. Following incubation, FACS analysis was performed on co-cultured cells and expression of co-stimulatory receptors; 4-1BB and OX-40 on (A) OT-I $CD8^+$ T cells and (B) OT-II $CD4^+$ T cells was evaluated. Experiment was performed in quadruplicate and is representative of 1-2 independent repeats. Data is presented as mean±SEM. P values represent one way-ANOVA and post-hoc Fisher's LSD tests. *P<0.05, **P<0.01, P<0.001, ****P<0.0001.

A CD8+ T cell frequency

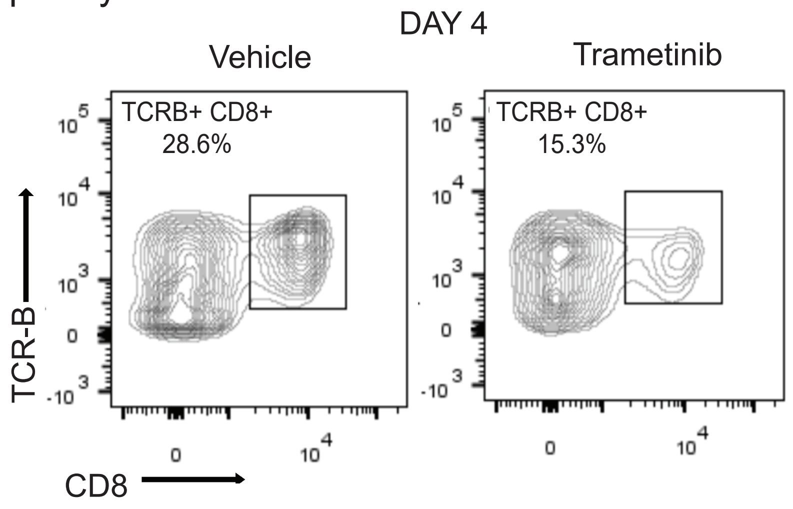

B Effector Function - CD8+ IFNγ

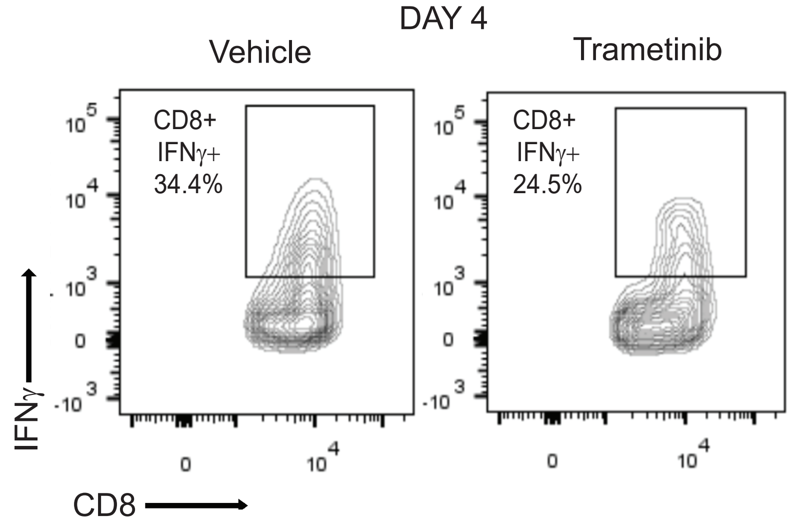

C Proliferation - CD8+ KI67

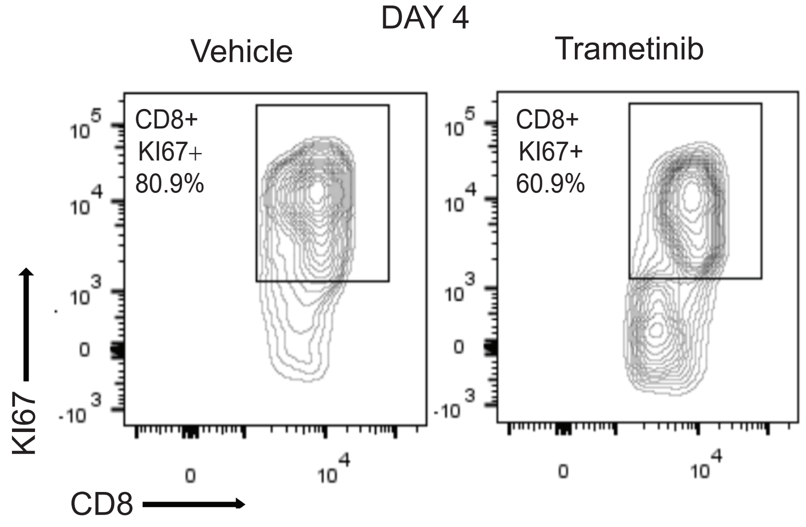

Supplementary Figure 3. Trametinib treatment effects CD8 T cell frequencies and function early in treatment response. Representative FACS plots are shown for day 4 data presented in Figure 3. TILs were restimulated with PMA (50 ng/ml) and ionomycin (1 µg/ml) for 4 hours in the presence of GolgiPlug/ GolgiStop. (A) $CD8^+$ T cell frequencies, (B) $CD8^+$ effector functions (IFNγ) and (C) $CD8^+$ proliferation (Ki67).

A NK Cells

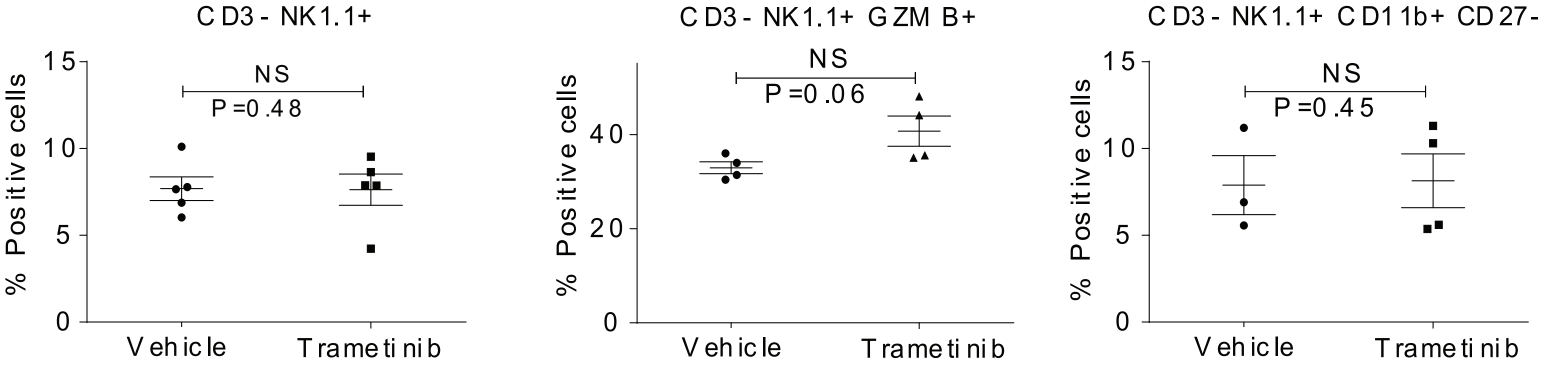

B NK T cells

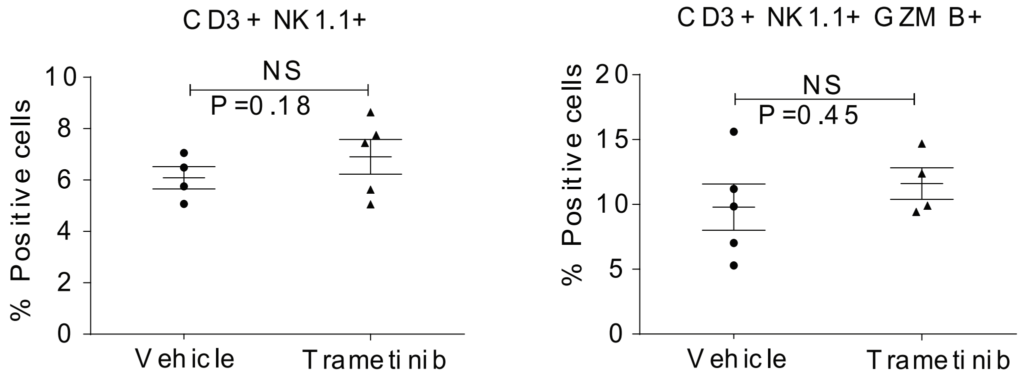

Supplementary Figure 4. Trametinib treatment has no impact on NK cell and NK T cell frequency and function. Mice (n=5 per group) bearing established AT3ova tumors were treated with vehicle (PEG 400/solutol), trametinib (1mg/kg/daily). Changes in NK and NK T ($CD3^{+}$ $NK1.1^{+}$) cell populations were determined *ex vivo by* FACS analysis 4 days post treatment for (A) NK cell frequency ($CD3^{-}$ $NK1.1^{+}$), maturation ($CD3^{-}$ $NK1.1^{+}$ $CD11b^{+}$ $CD27^{-}$) and granzyme B expression ($CD3^{-}$ $NK1.1^{+}$ GZM B^{+}) and (B) NK T cell frequency ($CD3^{+}$ $NK1.1^{+}$), and granzyme B expression ($CD3^{+}$ $NK1.1^{+}$ GZM B^{+}). Data is expressed as % of positive cells ± SEM. P values represent unpaired, two-tailed students' t-tests. *P<0.05, **P<0.01,*** P<0.001, ****P<0.0001.

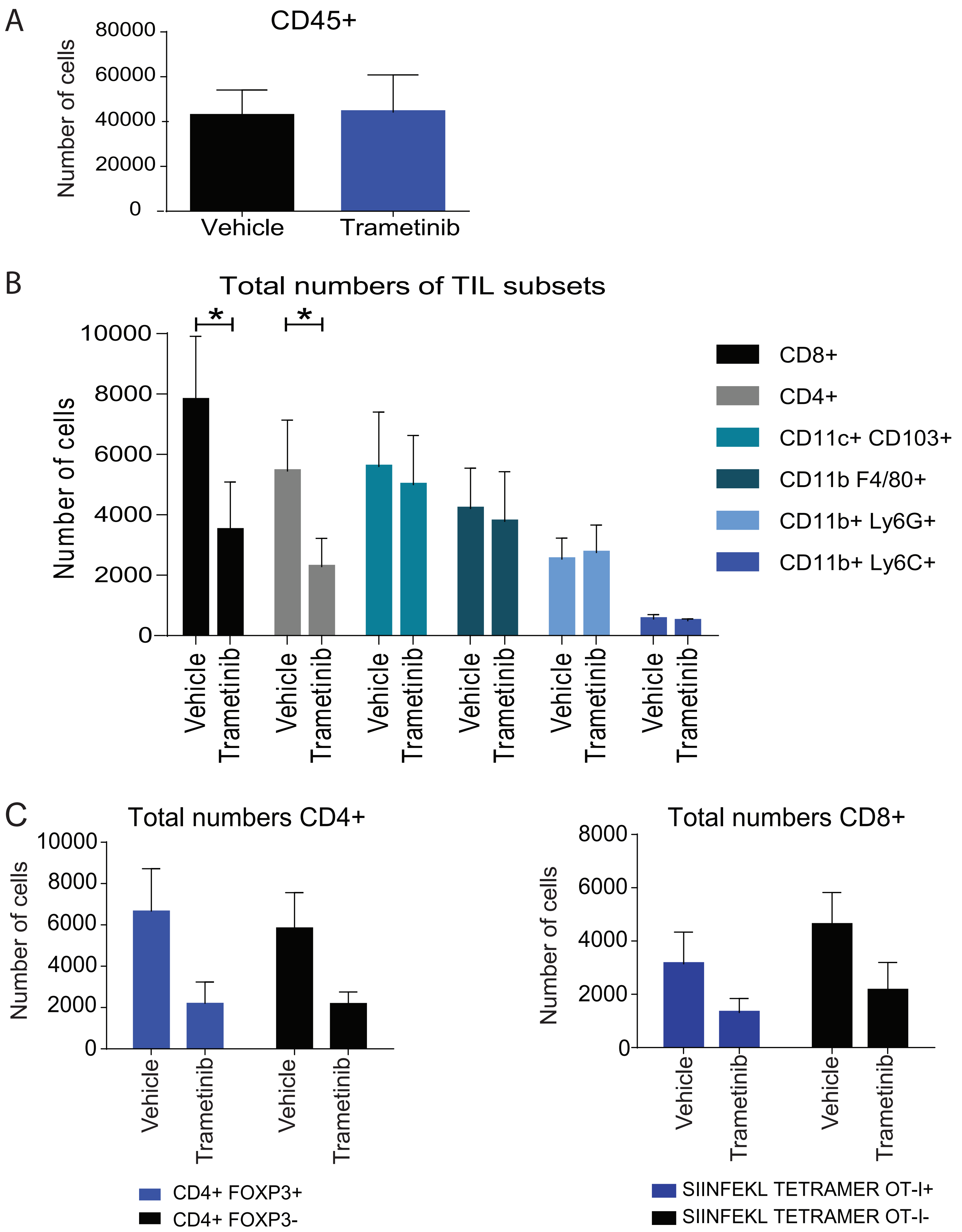
A
CD45+
Number of cells
80000
60000
40000
20000
0
Vehicle
Trametinib
B
Total numbers of TIL subsets
*
*
10000
8000
6000
4000
2000
0
Number of cells
CD8+
CD4+
CD11c+ CD103+
CD11b F4/80+
CD11b+ Ly6G+
CD11b+ Ly6C+
Vehicle
Trametinib
Vehicle
Trametinib
Vehicle
Trametinib
Vehicle
Trametinib
Vehicle
Trametinib
Vehicle
Trametinib
C
Total numbers CD4+
10000
8000
6000
4000
2000
0
Number of cells
Vehicle
Trametinib
Vehicle
Trametinib
CD4+ FOXP3+
CD4+ FOXP3-
Total numbers CD8+
8000
6000
4000
2000
0
Number of cells
Vehicle
Trametinib
Vehicle
Trametinib
SIINFEKL TETRAMER OT-I+
SIINFEKL TETRAMER OT-I-

Supplementary Figure 5. Trametinib treatment specifically effects T cell frequencies while immunosuppressive macrophage and myeloid derived suppressor subset cell numbers are unchanged over time. (A) Absolute numbers of CD45+ cells in vehicle and trametinib (n=6 mice/group) treated tumors at day 4 of treatment, quantified using counting beads. (B) Absolute numbers of $CD8^+$, $CD4^+$ T cells, DCs ($CD11c^+$ $CD103^+$), myeloid derived suppressor cells ($CD11b^+$ $Ly6G^+$/$CD11b^+$ $Ly6C^+$) and macrophage ($CD11b^+$ $F4/80^+$) in vehicle and trametinib treated tumors at day 4 of treatment. (C) Absolute numbers of total $CD4^+$; $CD4^+$ $FOXP3^-$/$FOXP3^+$ Tregs and total $CD8^+$; tetramer positive (SIINFEKL) and tetramer negative $CD8^+$ T cells at day 4 of treatment. Data is presented as mean ± SEM. Representatives from two independent repeats are shown. P values represent unpaired t-tests, post-hoc Fishers' LSD tests. *P<0.05, **P<0.01, *** P<0.001, ****P<0.0001.

A Mutational status of AT3 & 4T1

	Cell Line	Gene	Gene Description	Chromosome number	Position	Reference	Alteration	Variant type	Consequence
**Ras**									
	**4T1.9**	Rragc	Ras-related GTP binding C	4	123935646	T	C	Single nucleotide variation	Missense variant
**Negative pathway regulators**									
	**4T1.9**	Nf1	Neurofibromatosis 1	11	79441703	G	C	Single nucleotide variation	Splice donor variant
	**4T1.9**	Dusp1	Dual specificity phosphatase 1	17	26508254	A	G	Single nucleotide variation	Missense variant
	**4T1.9**	Dusp5	Dual specificity phosphatase 5	19	53529430	G	A	Single nucleotide variation	Missense variant
**Ras**									
	**AT3**	Rasl2-9	RAS-like, family 2, locus 9	7	5125916	C	T	Single nucleotide variation	Missense variant
**ERK**									
	**AT3**	Mapk3	Mitogen-activated protein kinase 3	7	126759736	G	A	Single nucleotide variation	Missense variant
**Negative pathway regulators**		(ERK1)							
	**AT3**	Dusp4	Dual specificity phosphatase 4	8	34807807	GGCGGCA	G	Deletion	Inframe deletion
	**AT3**	Dusp4	Dual specificity phosphatase 4	8	34807820	GCGGCAA	G	Deletion	Inframe deletion
	**AT3**	Dusp4	Dual specificity phosphatase 4	8	34807925	G	T	Single nucleotide variation	Missense variant

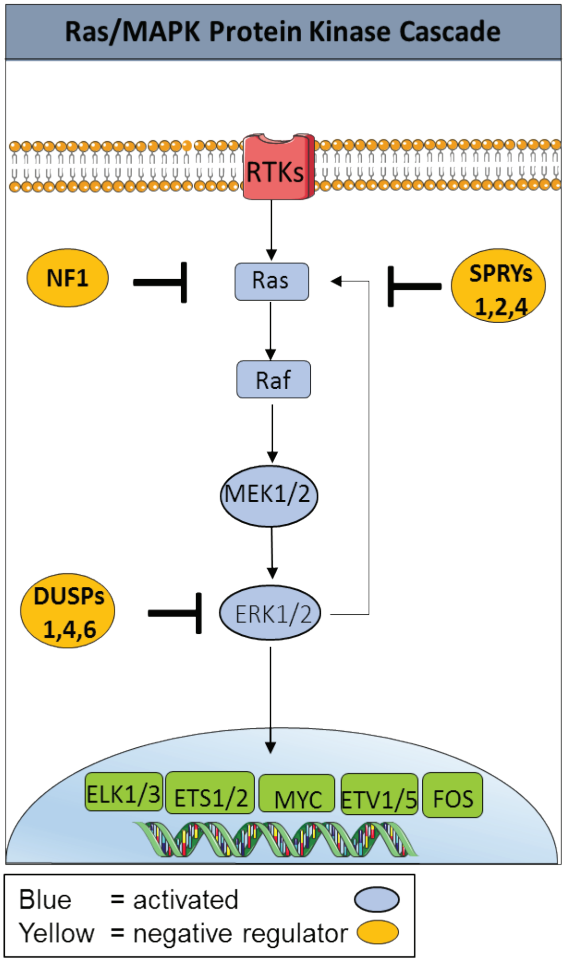

B Gene Set Enrichment Analysis - MEK Signature

AT3ova

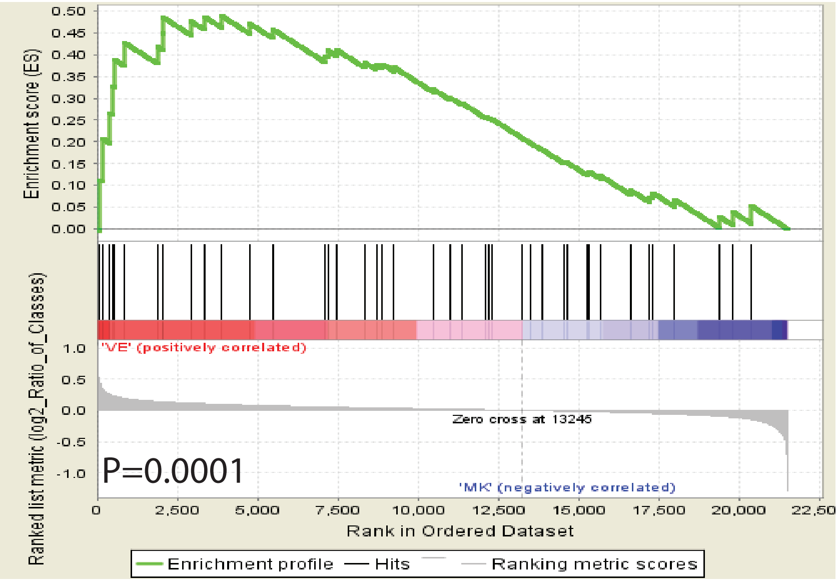

4T1Ch9

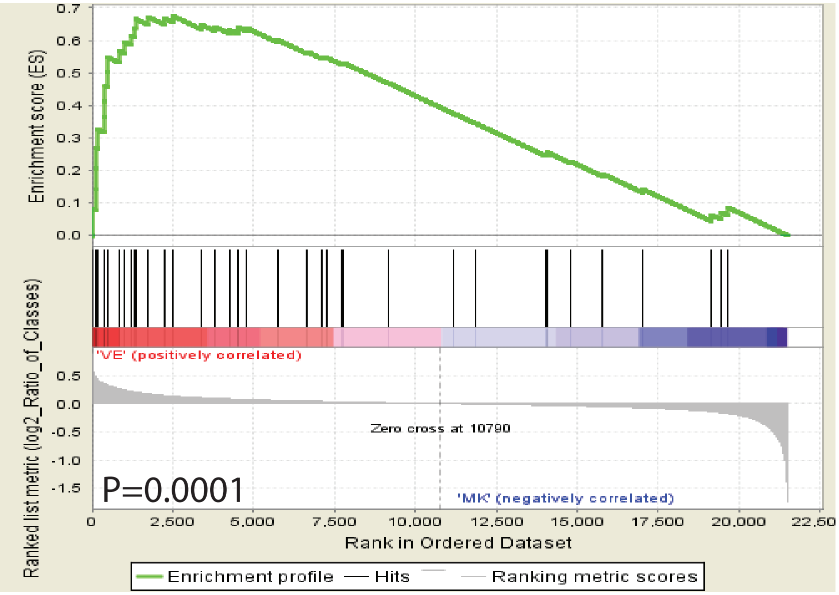

Supplementary Figure 6. Mutational status and MEK signature in the AT3ova and 4T1Ch9 cell lines. (A) Mutational status of the 4T1Ch9 and AT3 cell lines following whole exome sequencing analysis. (B) GSEA MEK signature of the 4T1Ch9 and AT3ova via *ex vivo* tumor analysis of baseline expression.

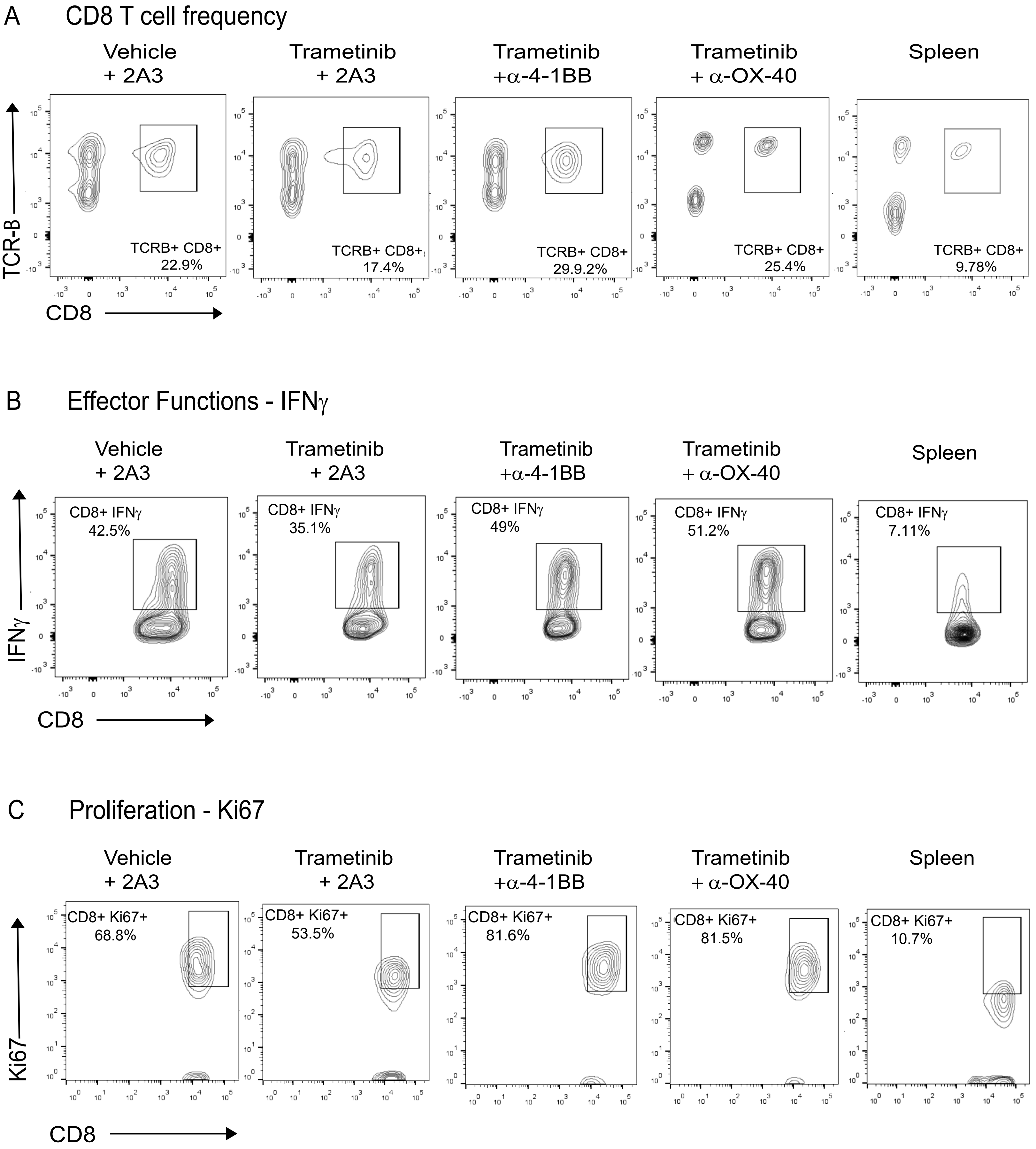
A
CD8 T cell frequency
Vehicle + 2A3
Trametinib + 2A3
Trametinib +α-4-1BB
Trametinib + α-OX-40
Spleen
TCR-B
CD8
TCRB+ CD8+ 22.9%
TCRB+ CD8+ 17.4%
TCRB+ CD8+ 29.9.2%
TCRB+ CD8+ 25.4%
TCRB+ CD8+ 9.78%
B
Effector Functions - IFNγ
Vehicle + 2A3
Trametinib + 2A3
Trametinib +α-4-1BB
Trametinib + α-OX-40
Spleen
IFNγ
CD8
CD8+ IFNγ 42.5%
CD8+ IFNγ 35.1%
CD8+ IFNγ 49%
CD8+ IFNγ 51.2%
CD8+ IFNγ 7.11%
C
Proliferation - Ki67
Vehicle + 2A3
Trametinib + 2A3
Trametinib +α-4-1BB
Trametinib + α-OX-40
Spleen
Ki67
CD8
CD8+ Ki67+ 68.8%
CD8+ Ki67+ 53.5%
CD8+ Ki67+ 81.6%
CD8+ Ki67+ 81.5%
CD8+ Ki67+ 10.7%

Supplementary Figure 7. Agonist immunotherapy rescues T cell effector functions in the presence of MEK inhibition. Representative FACS plots are shown for day 4 of combination therapies for data presented in Figure 8. TILs were restimulated with PMA (50 ng/ml) and ionomycin (1 µg/ml) for 4 hours in the presence of GolgiPlug/ GolgiStop. (A) $CD8^+$ T cell frequencies, (B) CD8+ effector functions (IFNγ), (C) $CD8^+$ effector functions (TNFα) and (D) $CD8^+$ proliferation (Ki67).

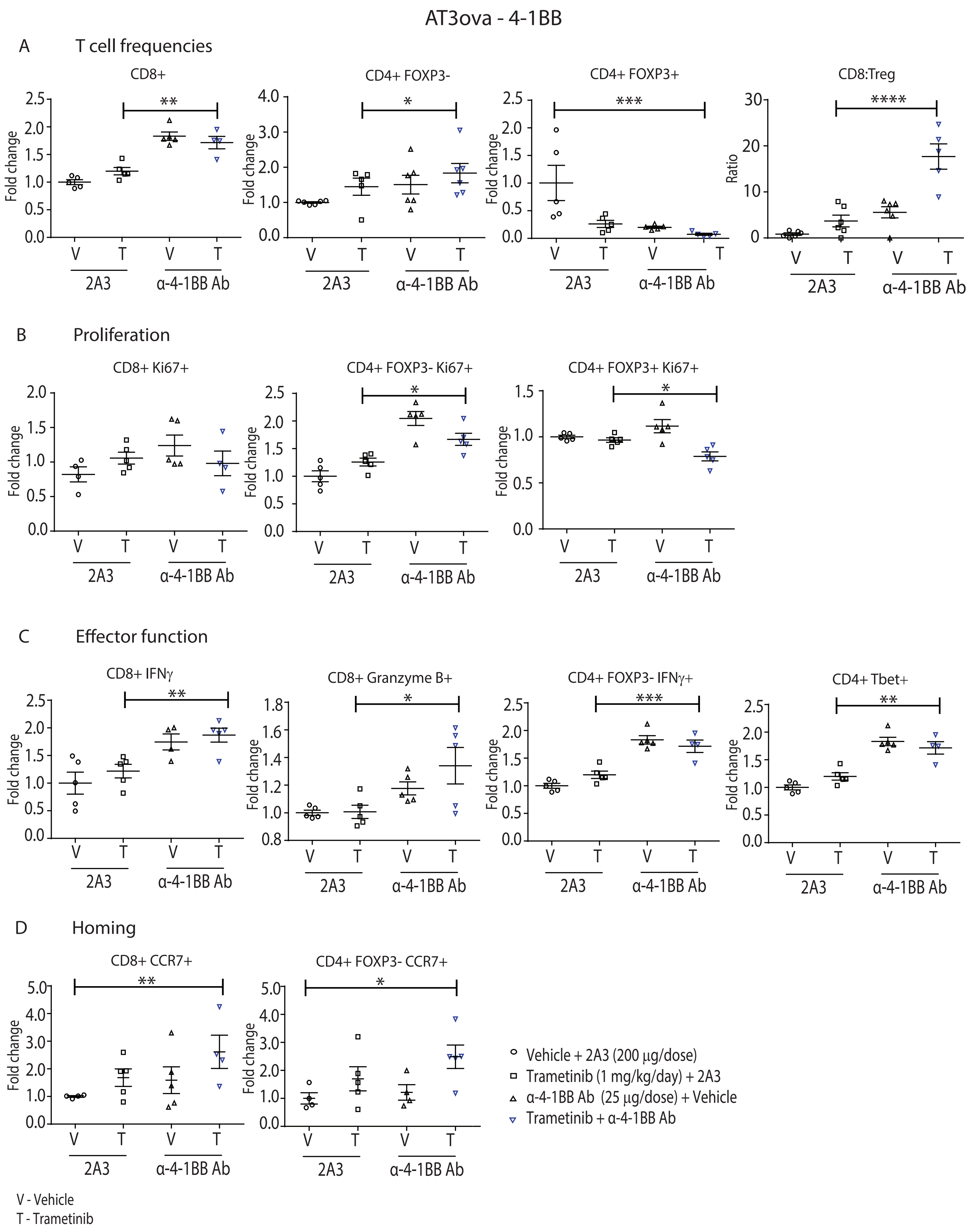
AT3ova - 4-1BB
A T cell frequencies
CD8+
CD4+ FOXP3-
CD4+ FOXP3+
CD8:Treg
Fold change
Ratio
B Proliferation
CD8+ Ki67+
CD4+ FOXP3- Ki67+
CD4+ FOXP3+ Ki67+
C Effector function
CD8+ IFNγ
CD8+ Granzyme B+
CD4+ FOXP3- IFNγ+
CD4+ Tbet+
D Homing
CD8+ CCR7+
CD4+ FOXP3- CCR7+
V
T
2A3
α-4-1BB Ab
Vehicle + 2A3 (200 μg/dose)
Trametinib (1 mg/kg/day) + 2A3
α-4-1BB Ab (25 μg/dose) + Vehicle
Trametinib + α-4-1BB Ab
V - Vehicle
T - Trametinib

Supplementary Figure 8. Trametinib and anti-4-1BB agonist combination therapy modulates TIL subsets in the AT3ova immunogenic TNBC model in the late response phase in vivo*.** Mice (n=5 per group) bearing established AT3ova tumors were treated with vehicle (PEG 400/solutol), trametinib (1mg/kg/daily) plus isotype control antibody IP injection (2A3; 200ug/dose) and either anti-4-1BB antibody (3H3;25ug/dose) IP injection on day 0, 4, 8, 12 or combination of trametinib and agonist antibody. Changes in TIL populations were determined *ex vivo by* FACS analysis 14 days post treatment for (A) TIL frequencies, (B) proliferation, (C) effector function and (D) homing. Values were normalized to vehicle controls in each experiment and data is expressed as fold change ± SEM for the number of positive cells. P values represent one way-ANOVA, post-hoc Fisher's LSD tests. *P<0.05, **P<0.01, P<0.001, ****P<0.0001.

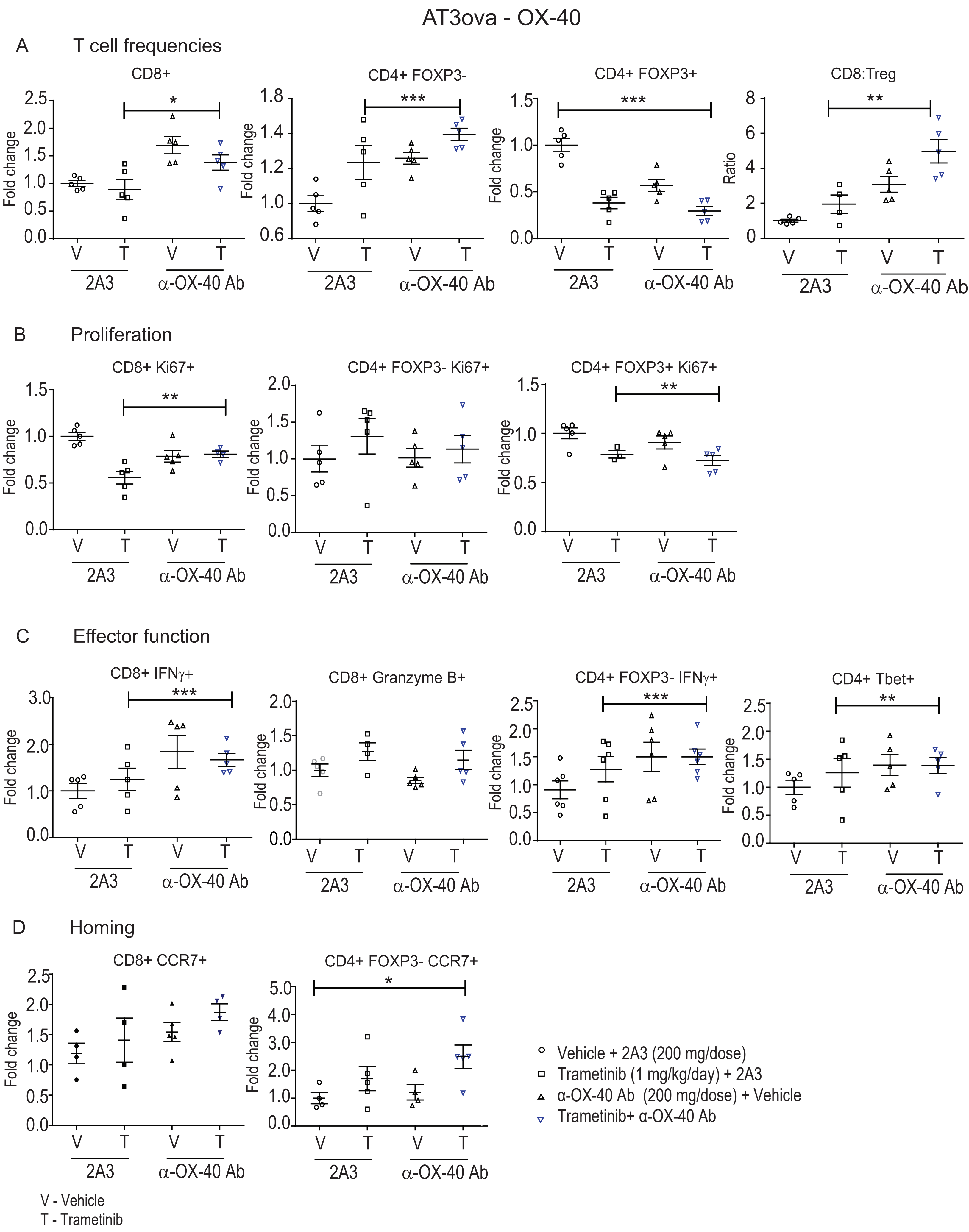
AT3ova - OX-40
A T cell frequencies
CD8+
CD4+ FOXP3-
CD4+ FOXP3+
CD8:Treg
Fold change
Ratio
*
***
**
V
T
2A3
α-OX-40 Ab
B Proliferation
CD8+ Ki67+
CD4+ FOXP3- Ki67+
CD4+ FOXP3+ Ki67+
C Effector function
CD8+ IFNγ+
CD8+ Granzyme B+
CD4+ FOXP3- IFNγ+
CD4+ Tbet+
D Homing
CD8+ CCR7+
CD4+ FOXP3- CCR7+
Vehicle + 2A3 (200 mg/dose)
Trametinib (1 mg/kg/day) + 2A3
α-OX-40 Ab (200 mg/dose) + Vehicle
Trametinib+ α-OX-40 Ab
V - Vehicle
T - Trametinib

Supplementary Figure 9. Trametinib and anti-OX-40 agonist combination therapy modulates TIL subsets in the AT3ova immunogenic TNBC model in the late response phase in vivo**.** Mice (n=5 per group) bearing established AT3ova tumors were treated with vehicle (PEG 400/solutol), trametinib (1mg/kg/daily) plus isotype control antibody IP injection (2A3; 200ug/dose) and either anti-OX-40 antibody (OX-86;200ug/dose) IP injection on day 0, 4, 8, 12 or combination of trametinib and agonist immunotherapy. Changes in TIL populations were determined *ex vivo by* FACS analysis 14 days post treatment for (A) TIL frequencies, (B) proliferation, (C) effector function and (D) homing. Values were normalized to vehicle controls in each experiment and data is expressed as fold change ± SEM for the number of positive cells. P values represent one way-ANOVA, post-hoc Fisher's LSD tests. *P<0.05, **P<0.01,*** P<0.001, ****P<0.0001.

A Pathway Analysis - Mouse

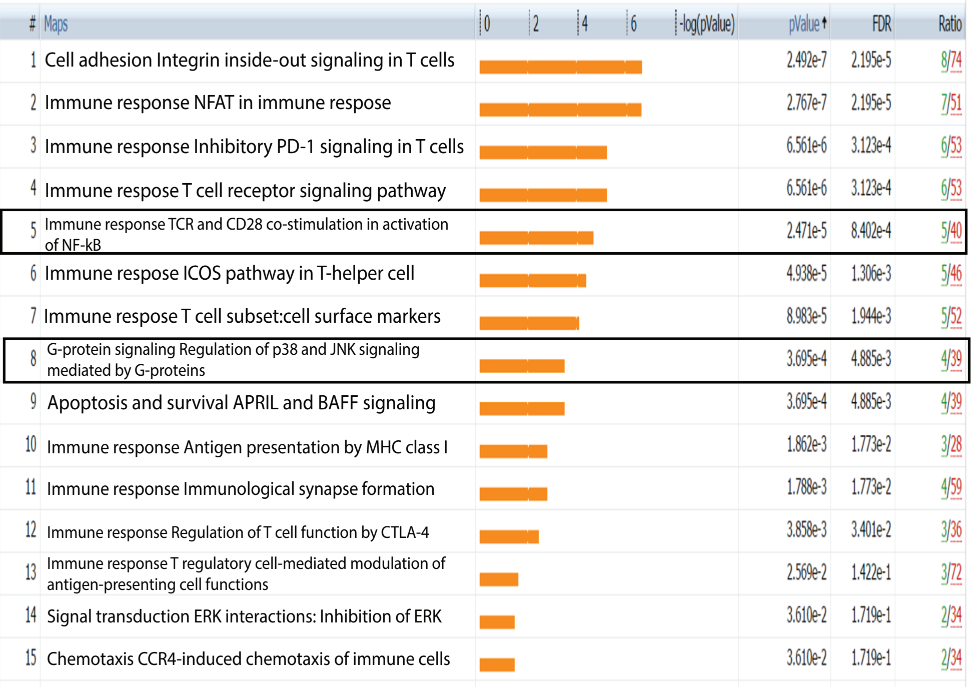

#	Maps	0 2 4 6 -log(pValue)	pValue	FDR	Ratio
1	Cell adhesion Integrin inside-out signaling in T cells		2.492e-7	2.195e-5	8/74
2	Immune response NFAT in immune respose		2.767e-7	2.195e-5	7/51
3	Immune response Inhibitory PD-1 signaling in T cells		6.561e-6	3.123e-4	6/53
4	Immune respose T cell receptor signaling pathway		6.561e-6	3.123e-4	6/53
5	Immune response TCR and CD28 co-stimulation in activation of NF-kB		2.471e-5	8.402e-4	5/40
6	Immune respose ICOS pathway in T-helper cell		4.938e-5	1.306e-3	5/46
7	Immune respose T cell subset:cell surface markers		8.983e-5	1.944e-3	5/52
8	G-protein signaling Regulation of p38 and JNK signaling mediated by G-proteins		3.695e-4	4.885e-3	4/39
9	Apoptosis and survival APRIL and BAFF signaling		3.695e-4	4.885e-3	4/39
10	Immune response Antigen presentation by MHC class I		1.862e-3	1.773e-2	3/28
11	Immune response Immunological synapse formation		1.788e-3	1.773e-2	4/59
12	Immune response Regulation of T cell function by CTLA-4		3.858e-3	3.401e-2	3/36
13	Immune response T regulatory cell-mediated modulation of antigen-presenting cell functions		2.569e-2	1.422e-1	3/72
14	Signal transduction ERK interactions: Inhibition of ERK		3.610e-2	1.719e-1	2/34
15	Chemotaxis CCR4-induced chemotaxis of immune cells		3.610e-2	1.719e-1	2/34

Gene set enrichment analysis - Mouse

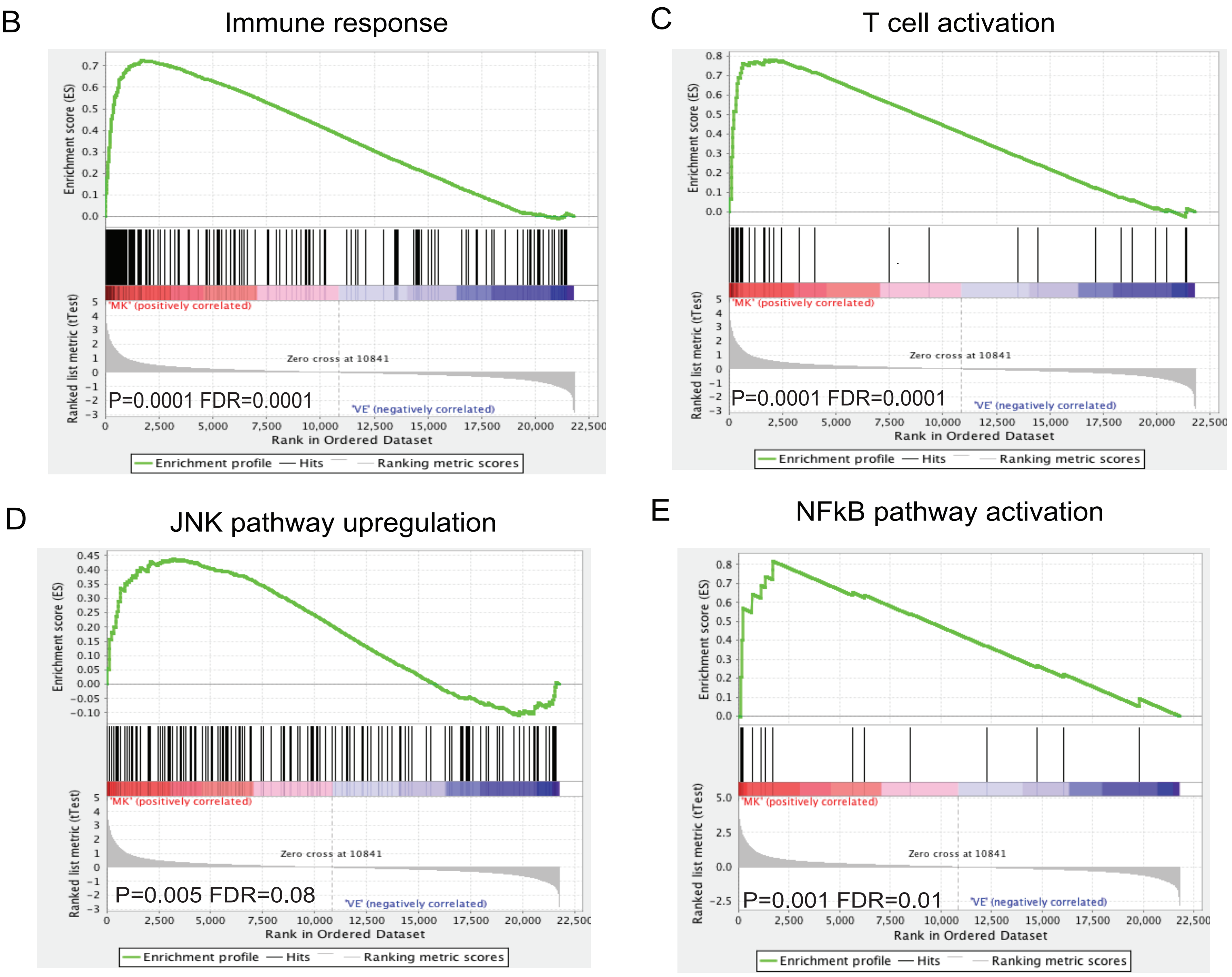

Supplementary Figure 10. Increased expression of chemokines and immune pathways following MEK inhibition in TNBCs**.** (A) Unbiased pathway analysis using GeneGo (molecular functions), based on top genes selected from differential expression analysis (log fold change) for an adjusted P value of <0.05 for MEKi versus vehicle treated 4T1Ch9 tumors. (B-E) GSEA analysis using the Broad Institute database of signatures using gene expression data from Affymetrix analysis of 4T1Ch9 *ex vivo* tumors. Positive correlation enrichment plots for (B) Immune response, (C) T cell activation, (D) JNK pathway and (E) NFκB pathway. Complete lists are provided in Supplementary Material.

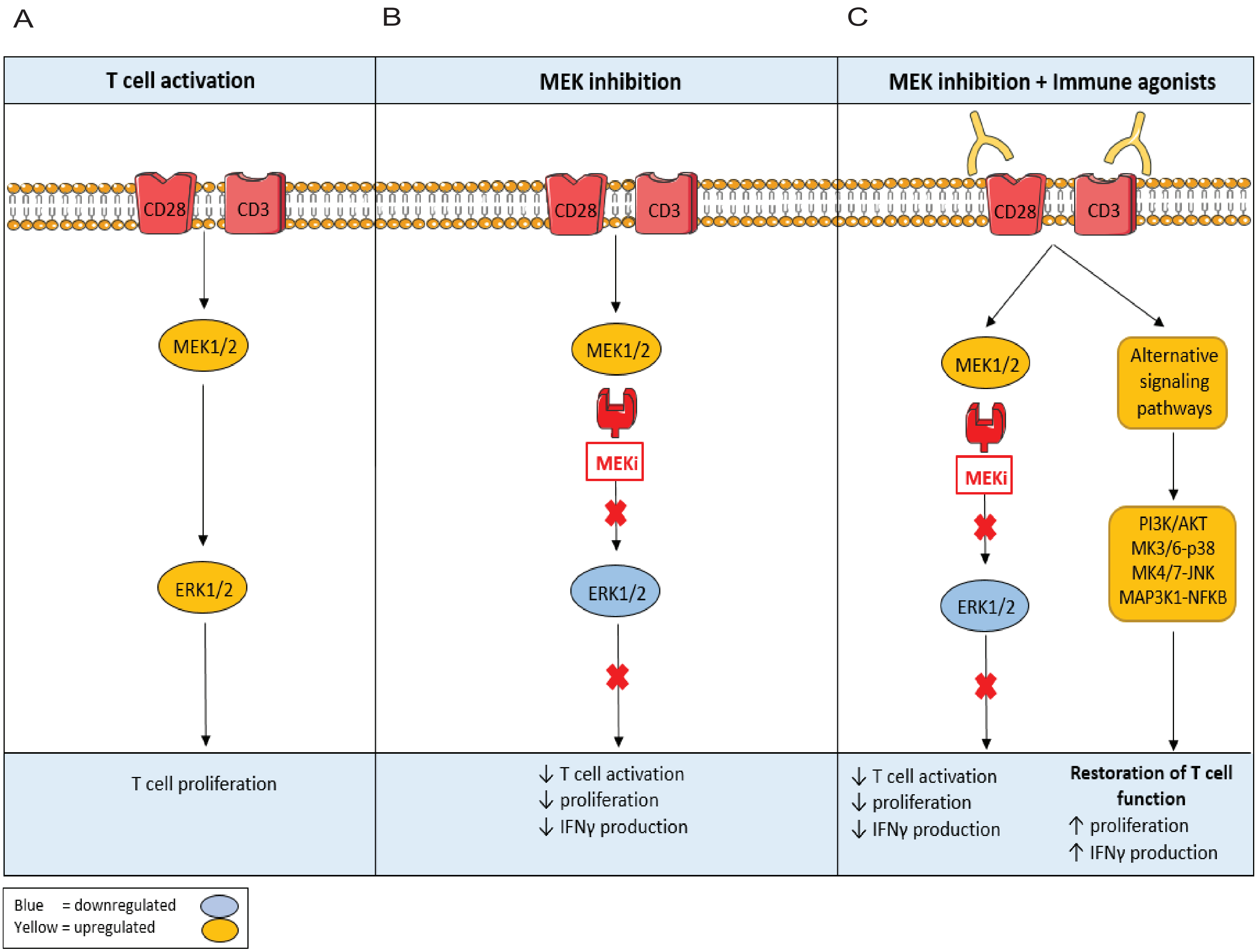

A
B
C
T cell activation
MEK inhibition
MEK inhibition + Immune agonists
CD28
CD3
CD28
CD3
CD28
CD3
MEK1/2
ERK1/2
MEK1/2
MEKi
ERK1/2
MEK1/2
MEKi
ERK1/2
Alternative signaling pathways
PI3K/AKT
MK3/6-p38
MK4/7-JNK
MAP3K1-NFKB
T cell proliferation
↓ T cell activation
↓ proliferation
↓ IFNγ production
↓ T cell activation
↓ proliferation
↓ IFNγ production
Restoration of T cell function
↑ proliferation
↑ IFNγ production
Blue = downregulated
Yellow = upregulated

***Supplementary Figure 11*. *Agonist antibodies rescue crucial T cell signaling through alternative pathways, independent of classical ERK1/2 activation*.** Proposed schematic of the mechanism for re-direction of signalling through alternative MAPK pathways to classical activation in T cells, with the addition of agonist antibodies following MEKi (MEK inhibition). (A) Classical activation in T cells following CD3 and CD28 co-stimulation, is inhibited following MEKi (B). (C) Activation of alternative MAPK and PI3K pathways; p38 (MK3/6), JNK (MK4/7) and NFκB (MAP3K1) following activation of the 4-1BB and OX-40 co-stimulatory receptors on T cells with α-4-1BB or α-OX-40 agonist immunotherapy, in the absence of classical MEK signaling due to MEKi.

Original Western Blots

Western Blots - Mouse (Main Figure 10B)

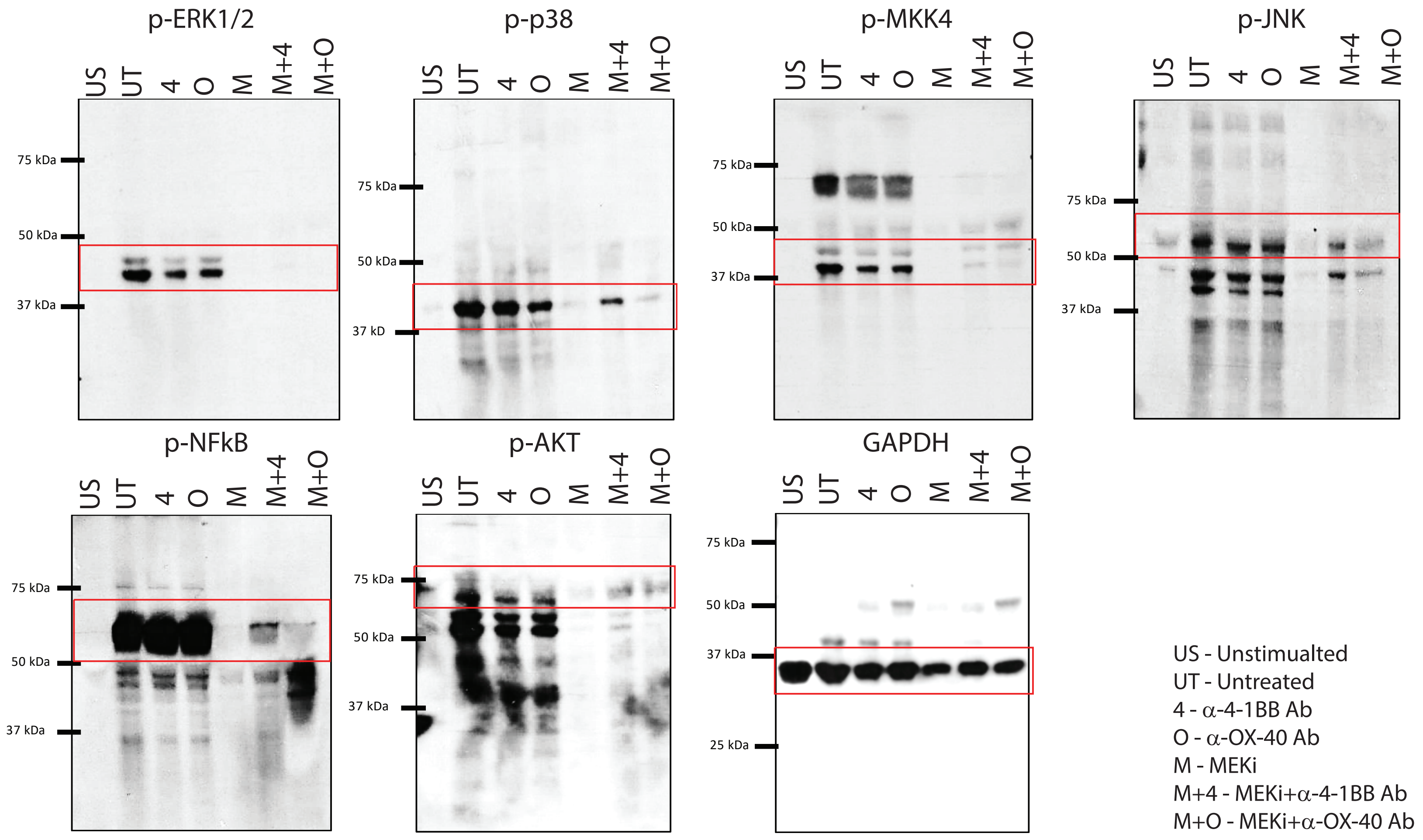

Western Blots - Human (Main Figure 10B)

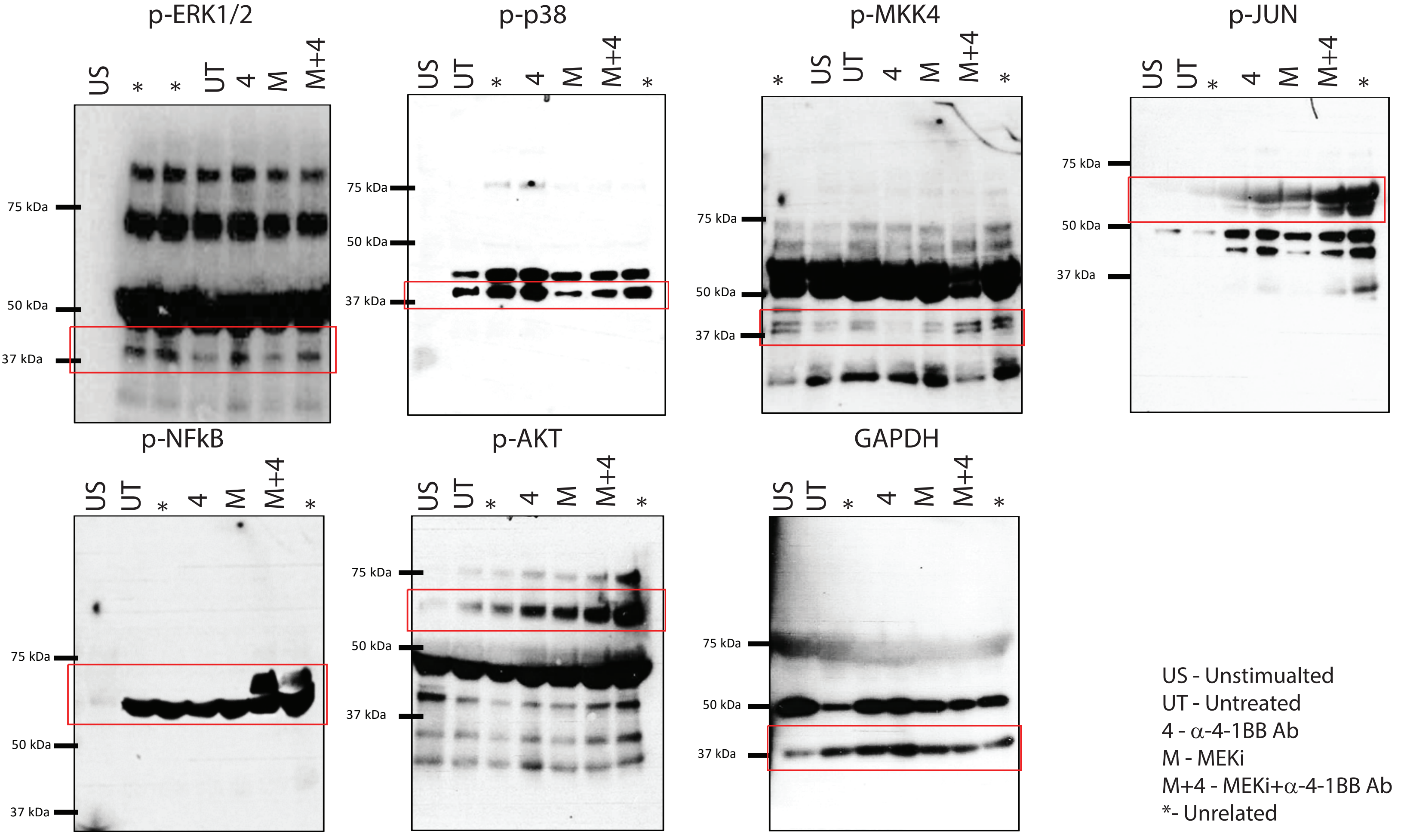

Supplementary Figure 12. T cell signaling restoration occurs via alterative ERK independent MAPK pathway activation. Mouse and human T cell signaling was analysed using purified $CD8^+$ mouse T cells isolated from mouse spleen and activated for 16 h with α-CD3 (1 µg/ml) antibody. Treatment groups include: unstimulated or stimulation with α-CD3/CD28 antibody (0.5 µg/ml in mouse), trametinib (10nM), α-4-1BB (50 µg/ml) or α-OX-40 (50 µg/ml) antibody alone, or combination of trametinib and agonist antibody, for an incubation period of 72 h. Human T cell signaling was analysed using purified $CD45RA^+$ $CD8^+$ human T cells isolated from human PBMCs and activated for 16 h with α-CD3 (OKT3; 1 µg/ml) antibody. Treatment groups were as above, dosed for an incubation period of 5 min. Western Blot analysis was performed for phosphorylated proteins of T cell signaling pathways; p-ERK (42/44k Da), p-p38 (43 kDa), p-MKK3/6 (38/40 kDa), p-MKK4 (44 kDa), p-AKT (60 kDa), p-JNK (54 kDa), c-JUN (48 kDa), p-NFκB (65 kDa), and GAPDH (40 kDa).
